# Factors influencing the online sale of prescription medication in Iran: insights from a qualitative content analysis

**DOI:** 10.1186/s12913-026-15338-9

**Published:** 2026-08-18

**Authors:** Amin Assarian, Fatemeh Soleymani, Akbar Abdollahiasl, Manuchehr Bashirynejad

**Affiliations:** 1 Department of Pharmacoeconomics and Pharmaceutical Administration, School of Pharmacy, Tehran University of Medical Sciences, 16 Azar Street, Tehran, Iran; 2 Research Center for Rational Use of Drugs, Tehran University of Medical Sciences, Tehran, Iran; 3 Research Center for Pharmacoeconomics and Pharmaceutical Administration, Institute of Pharmaceutical Sciences, Tehran University of Medical Sciences, Tehran, Iran

**Keywords:** Prescription drugs, Pharmaceutical services online, Qualitative research, Health policy

## Abstract

**Background:**

The digital transformation in healthcare has presented both opportunities and challenges for the online sale of prescription medications, particularly in developing countries such as Iran. Online pharmacies have the potential to enhance access, convenience, and affordability; however, the absence of a robust regulatory framework and concerns about patient safety, counterfeit medications, and market monopolization pose significant obstacles. This study aims to explore the critical factors influencing the online sale of prescription medications in Iran to inform policy and practice.

**Methods:**

A qualitative study was conducted using a conventional content analysis approach. Seventeen semi-structured interviews were carried out with stakeholders, including pharmacists, legal experts, and policymakers, between July and October 2024. Themes and sub-themes were identified using an inductive approach, and data were analyzed using MAXQDA software. The trustworthiness of the data was ensured through credibility, dependability, confirmability, and transferability measures.

**Results:**

The analysis identified six main themes and 20 sub-themes influencing the online sale of prescription medication in Iran: Legal and Regulatory Challenges, Ethical Considerations, The Role of the Pharmacist, Patient Behavior and Safety, Technology and Infrastructure, and Economic Implications. Key barriers included regulatory ambiguities, ethical concerns such as data privacy and equity, and limitations in technological infrastructure. Participants proposed solutions, including establishing clear legal frameworks, multidisciplinary oversight panels, and secure technological platforms.

**Conclusion:**

The study underscores the need for comprehensive legal and regulatory frameworks, ethical safeguards, and robust technological infrastructure to support the safe and equitable implementation of online prescription medication sales in Iran. These efforts should address malpractice risks, enhance patient trust, and ensure equitable access, paving the way for a sustainable digital transformation in pharmaceutical services.

**Supplementary Information:**

The online version contains supplementary material available at https://doi.org/10.1186/s12913-026-15338-9.

## Introduction

The rapid advancement of digital technology has transformed various sectors, including healthcare, where e-commerce has emerged as a crucial component in medication distribution. The online sale of prescription medication offers several advantages, such as increased accessibility, convenience, and the potential for cost savings [1]. However, these benefits also entail inherent risks, including regulatory challenges and concerns related to patient safety and counterfeit medications [2].

Globally, the regulation and implementation of online pharmacies vary significantly. In high-income countries such as the United States and Canada, certified online pharmacies operate under stringent regulatory frameworks, such as the Verified Internet Pharmacy Practice Sites (VIPPS) accreditation. The European Union mandates a common logo for legal online pharmacies to assist consumers in distinguishing them from illegal sellers [2]. However, challenges persist, particularly with cross-border sales and the monitoring of unregulated platforms. A World Health Organization report estimates that over 50% of medicines purchased online from illegal websites may be counterfeit or substandard [3].

In developing countries, the landscape for online pharmacies varies widely. For example, countries such as India and Brazil have experienced significant growth in their online pharmacy sectors but continue to face regulatory and enforcement challenges [4, 5]. In Iran, the online pharmacy landscape is evolving but remains underdeveloped compared to many Western nations. Currently, the legal framework governing the sale of online prescription medications is ambiguous, primarily permitting the sale of non-prescription items, such as dietary supplements and cosmetics [6].

This regulatory gap raises concerns about the safety and efficacy of medications purchased through online platforms, especially in a context where counterfeit medications represent a significant threat [7]. Research indicates that a lack of regulatory oversight can lead to the proliferation of unauthorized online pharmacies, further increasing the risk of substandard medications [8].

The dominance of large e-commerce platforms has further complicated the landscape, as these platforms often operate with significant market power, offering aggressive discounts, extensive advertising, and undermining smaller, community-based pharmacies [9, 10].

Despite growing attention to this issue in public health and policy discussions, there remains a lack of empirical data examining the drivers, barriers, and consequences of online prescription medication sales in Iran. While some quantitative surveys have explored consumer behavior in online health purchases, no published qualitative studies have investigated stakeholder perspectives within the Iranian context. This gap limits policymakers’ ability to design informed, evidence-based interventions.

This study aims to address this gap by exploring the experiences and perspectives of pharmacists, legal experts, and policymakers concerning the online sale of prescription medications in Iran. Employing a qualitative content analysis approach, the study seeks to identify key regulatory, ethical, and infrastructural factors influencing this phenomenon.

## Methods

### Study design

This study utilized a conventional qualitative content analysis approach to explore the perspectives of key stakeholders regarding the online sale of prescription medications in Iran. An inductive, data-driven methodology was employed to allow themes to emerge directly from participant narratives without applying a predetermined theoretical framework [11–13]. This approach was chosen to identify underlying patterns and relationships and to offer a nuanced understanding of this complex issue.

### Participants

A purposive sampling strategy was employed to select participants who had relevant expertise and experience in the online sale of prescription medications. A total of 17 participants were interviewed. They were identified and approached via phone or professional introductions within academic and institutional networks. Specifically, three stakeholder groups were targeted:

- Managers of online pharmacies with over five years of experience overseeing both digital and traditional brick-and-mortar pharmacies. Their expertise provided important insights into the day-to-day operations of online pharmacies.
- Legal professionals who have worked on at least two cases related to pharmacy law. They offered essential views on the legal framework affecting online pharmacies.
- Policymakers whose decisions significantly influence the online pharmacy industry, providing valuable perspectives on the regulatory environment and possible future changes in policy. They were senior officials from the Ministry of Health, regulatory bodies overseeing pharmaceutical services, and representatives from professional pharmacy associations. These groups provided insights into operational, legal, and policy dimensions of online pharmacy practices. The only exclusion criterion was the participant’s decision to leave the research during the study.

### Data collection

Data were collected from 20 July to 21 October 2024 via semi-structured, in-depth, face-to-face interviews conducted in private settings such as workplaces or institutional offices. During each interview, field notes were taken by the interviewer to capture non-verbal cues, contextual details, and immediate impressions to complement the audio-recorded data.

The interviews were guided by an interview protocol consisting of open-ended questions designed to elicit participants’ perspectives on factors influencing the online sale of prescription medications. The guide was pilot-tested with a pharmacist and a legal expert to assess the clarity, relevance, and comprehensiveness of the questions, and the pilot interviews were excluded from analysis. Minor adjustments were made during the early stages of data collection in response to participants’ feedback and the interviewer’s reflection.

All interviews were conducted by the first author, a pharmacist based in Tehran, who had prior training in qualitative interviewing. Interviews continued until data saturation was reached after 15 interviews, which was defined as the point at which no new themes or sub-themes emerged from subsequent interviews. To avoid false saturation, two supplementary interviews were conducted. These were carried out using the same semi-structured interview guide and analyzed using the same inductive content analysis approach.

Following the approach by Saunders et al. (2018), saturation was recognized when additional interviews produced no further meaningful variation or novel insights related to the study objectives [14]. Interviews lasted 45 to 60 min each. The interview guide was specifically developed for this study, informed by previous research on the challenges and practices of online pharmacy sales [4, 15–5]. English version of the interview guide is available in Additional file 1.

Semi-structured interviews were selected because of their specific subject focus and the developed bilateral relationship between the researcher and interviewee. This approach provided good qualitative data; at the same time, it allows the researcher to maintain control in the data collection process [16].

### Data analysis

Data analysis commenced simultaneously with data collection, following the approach described by Graneheim and Lundman [17], to allow an iterative process between concept identification and data collection. Verbal transcription into a computer document was performed immediately after each interview. Then, transcripts were imported into MAXQDA software, version 12, for efficient data management. The primary researcher carefully reviewed the transcripts multiple times to gain a comprehensive understanding of the content.

The entire text of each interview was taken as a unit of analysis, and words, sentences, or paragraphs were considered as meaning units. These units were consolidated based on their content, forming condensed meaning units. Afterward, these condensed meaning units were abstracted, labeled, and consequently transformed into codes. This included both in-vivo coding (using participants’ exact words) and researcher-generated codes based on interpretation. Next, codes were thoroughly examined, comparing similarities and differences, and subsequently categorized into various sub-themes and main themes. This classification allowed for a more profound comprehension of the themes and patterns within the data and, therefore, allowed for the emergence of key factors influencing the online sale of prescription medication in Iran.

This study adhered to the Consolidated Criteria for Reporting Qualitative Research (COREQ) guidelines to ensure transparency and rigor in reporting. A completed COREQ checklist is provided in Additional file 2.

### Trustworthiness

To ensure the trustworthiness of the study, the criteria of credibility, dependability, confirmability, and transferability were addressed [18]. Credibility was established through prolonged engagement and appropriate interaction with participants. To further enhance credibility, member checking was employed, whereby participants were provided with full transcripts of selected interviews and extracted codes for verification. Dependability was established through peer checking conducted by co-authors, who independently reviewed the coding structure and thematic categorization. Confirmability was strengthened by using a diverse sample with maximum variation. Lastly, transferability was promoted through the inclusion of direct quotes in the analysis.

To minimize analyst bias and enhance reflexivity during data analysis, the research team engaged in memo writing, peer debriefing, and code triangulation. Multiple researchers independently coded selected transcripts and collaboratively discussed discrepancies to ensure consistency and data-driven interpretation. Regular team discussions also fostered critical reflection on how the researchers’ professional roles and prior assumptions might have influenced the analytical process.

### Ethical consideration

The study adhered strictly to ethical standards and guidelines. Ethical approval was obtained from the Ethics Committee of Tehran University of Medical Sciences (ethics code: IR.TUMS.TIPS.REC.1402.064), and the research complied with national guidelines for medical research in Iran.

Before each interview, participants were provided with a detailed explanation of the study’s aims. Their written consent was obtained, confirming their willingness to participate and permission to record the interviews. Separate consent was obtained for each stage of the interview.

Participant privacy was protected by ensuring the confidentiality of their information and safeguarding their identities. Moreover, participants were granted access to the research findings upon request. These steps were taken to maintain ethical integrity and prioritize the confidentiality of all involved participants.

## Results

In this study, 17 eligible participants were interviewed. No prior relationship existed between the interviewers and interviewees. The participants’ ages ranged from 38 to 75 years, with 29% being female. Among them, 13 had expertise in pharmacy and played overlapping roles such as policymakers or legal professionals. Specifically, five participants were legal experts (one with a pharmacy background), and five served as policymakers. The demographic and professional details of the participants are summarized in Table 1.

**Table 1:** Demographic characteristics of study participants

Participant ID	Profession	Age range (years)	Years of Experience
PH#1	Pharmacist	40–49	22
PH#2	Pharmacist	70–79	45
PH#3	Pharmacist	70–79	49
LEG#4	Legal professional	50–59	26
PH#5	Pharmacist/ managing director of an online pharmacy	30–39	14
MOH#6	Pharmacist / Policymaker	50–59	21
MOH#7	Pharmacist / Policymaker	40–49	22
LEG#8	Pharmacist/ Legal professional	50–59	20
MOH#9	Pharmacist / Policymaker	50–59	18
PH#10	Pharmacist/ Managing director of an online pharmacy	40–49	10
PH#11	Pharmacist / Managing director of an online pharmacy	40–49	12
PH#12	Pharmacist / Managing director of an online pharmacy	40–49	24
LEG#13	Legal professional	50–59	26
MOH#14	Pharmacist / Policymaker	40–49	17
MOH#15	Pharmacist / Policymaker	50–59	21
LEG#16	Legal professional	50–59	28
LEG#17	Legal professional	50–59	26

During the interviews, participants discussed various aspects of online prescription medicine sales in Iran, including their perceived advantages, disadvantages, challenges, and concerns, as well as potential opportunities and threats. They also evaluated the effectiveness of current processes and systems used by online pharmacies, particularly in terms of safeguarding patient safety and ensuring the integrity of prescription verification. Additionally, participants proposed practical solutions to address the challenges identified in the distribution process of online medications. Finally, they examined the potential role of technology integration and interdisciplinary collaboration in improving the quality, safety, and accessibility of online pharmacy services.

Using conventional content analysis, the study identified several key factors influencing the online sale of prescription medications in Iran. From 98 initial codes, six major themes and 20 sub-themes were derived. The six main themes include “Legal and Regulatory Challenges,” “Ethical Considerations,” “The Role of the Pharmacist,” “Patient Behavior and Safety,” “Technology and Infrastructure,” and “Economic Implications” (Table 2).

**Table 2:** The themes extracted from factors influencing the online sale of prescription medication in Iran

Themes	Sub-themes	Examples of initial coding
Legal and Regulatory Challenges	Lack of Clear Guidelines	Current regulations only cover non-prescription items, creating a void for prescription medications, which causes confusion and operational challenges for pharmacies.
	Enforcement Challenges	Limited resources and lack of a regulatory body result in inconsistent oversight, leading to increased risk of misuse
	Legal Responsibility	Lack of accountability framework for medication errors in online sales; participants emphasize the need for liability protections for patients and pharmacists.
	Panel of Experts	Suggested interdisciplinary panel (pharmacists, IT, legal experts) to create regulatory guidelines for online pharmacies
Ethical Considerations	Prescription Forgery and Authenticity	High risk of prescription forgery in an online setting; security measures needed to ensure prescription authenticity.
	Fair Access and Equity	Concerns that online pharmacies may deepen healthcare inequalities; the importance of equitable access, especially for vulnerable groups.
	Marketing and Advertising Practices	Unregulated online marketing of prescription drugs could mislead consumers, creating ethical concerns.
	Logistics and Distribution Systems	Recommendation for pharmacies to manage logistics, or partner with licensed companies for secure, supervised delivery.
The Role of the Pharmacist	Provision of Pharmaceutical Advice	Online sales may bypass pharmacists’ advisory roles, impacting patient education; recommended integration of pharmacist consultations.
	Prescription Verification	Need for a robust system to verify prescriptions online, ensuring patients receive appropriate medications.
	Medication Quality and Safety Assurance	Ensuring quality and safety during delivery, especially for temperature-sensitive drugs; pharmacists essential in verifying product authenticity.
Patient Behavior and Safety	Patient Awareness and Education	Public lacks awareness of online pharmacy risks; educational initiatives recommended to promote safe online purchase practices.
	Privacy and Confidentiality	Strong data protection measures necessary to maintain patient trust; delivery systems should secure sensitive health information.
	Consumer Confidence	Consistent quality standards and verified prescription validation needed to enhance trust in online pharmacies.
Technology and Infrastructure	Effective Online Platforms	Comprehensive e-health databases needed for secure online prescription sales; user-friendly and accessible platforms critical for patient experience.
	Data Security and Privacy Protection	High risk of data breaches; stringent cybersecurity protocols are needed to safeguard patient information.
	Internet Access and Digital Inequality	Limited internet access, especially in rural areas, restricts online pharmacy reach, posing barriers to healthcare inclusivity.
Economic Implications	Pricing Structures	Disparities between online and in-person pharmacy prices may arise; fair pricing structures are essential for equitable access. Concerns about larger online platforms monopolizing the market, threatening the viability of smaller, independent pharmacies
	Drug Supply Chain	Shift to online sales disrupts traditional pharmaceutical supply chain, affecting logistics and inventory management
	Insurance Integration	Lack of insurance coverage for online purchases is a financial barrier; integration with insurance frameworks suggested to enhance affordability

Based on these themes, six sets of key policy and practice recommendations were extracted. These recommendations include the establishment of clear regulatory frameworks, ethical safeguards, enhanced pharmacist roles, public education initiatives, technological infrastructure development, and fair economic practices to support the safe and equitable implementation of online prescription medication services in Iran, as summarized in Table 3.

**Table 3:** Summary of key recommendations aligned with identified themes

Theme	Key Recommendations
Legal & Regulatory Challenges	Establish clear, unified regulations; create a centralized regulatory authority and a multidisciplinary oversight panel.
Ethical Considerations	Implement robust prescription verification systems; promote fair access policies; regulate ethical marketing and distribution practices.
Pharmacist’s Role	Integrate pharmacist–patient interaction features into online platforms; strengthen the pharmacist’s role in prescription verification and patient education.
Patient Behavior & Safety	Launch public awareness campaigns; ensure data privacy and confidentiality; build consumer trust through transparency.
Technology & Infrastructure	Develop user-friendly, secure e-pharmacy platforms; integrate with electronic health records and insurance systems; address digital inequality.
Economic Implications	Introduce fair pricing regulations; prevent monopolistic practices; integrate insurance coverage for online prescriptions.

### Legal and regulatory challenges

This theme included four subthemes: lack of clear guidelines, Enforcement Challenges, legal and responsibilities, and Panel of experts. A key issue raised by the majority of participants, especially policymakers, was the urgent need for transparent regulations and defined oversight mechanisms for the online sale of prescription medications.

#### Lack of clear guidelines

There was broad agreement among legal experts and policymakers that the regulatory gap surrounding prescription medicines sales online has left stakeholders uncertain and exposed

The lack of explicit policies for prescription medication has left both patients and pharmacies vulnerable. (PH#10)

Some participants pointed out that while ethical guidelines exist, they do not specifically address digital sales channels.

We developed and published a comprehensive pharmaceutical ethics document. However, to my knowledge, this document does not address the online sale of medications. (MOH#14)

Pharmacists noted the confusion this legal ambiguity creates in everyday operations, especially given the allowance of OTC products but not prescriptions.

Only supplements and cosmetic products are legally permitted for online sales. This has created a regulatory void for prescription medication, causing confusion among pharmacies. (PH#11)

Legal experts emphasized that this lack of specificity compromises safety and leaves critical aspects of the process unregulated.

Without specific guidelines, ensuring the safety of online medication distribution is challenging. (LEG#4)

#### Enforcement challenges

In addition to missing guidelines, participants expressed concern over fragmented and ineffective enforcement mechanisms. A centralized authority was seen as essential for ensuring compliance.

There is no transparent regulatory framework to govern the online sale of prescription medication in Iran. A central regulatory body could provide the necessary oversight and prevent abuse. (LEG#8)

This highlights the structural gaps in regulatory governance, which currently lack coordination across relevant bodies.

#### Legal and responsibility

Uncertainty around legal accountability in cases of online dispensing errors or harm emerged as a serious concern. Participants noted that existing laws are not only inconsistent but also leave critical questions unanswered.

Inconsistent oversight of online pharmacies leads to a lack of accountability and increased risks of abuse. (MOH#14)

Many existing laws are scattered and inconsistent, resulting in contradictions during enforcement. A unified and clear law is critically needed to regulate and monitor online medication sales. (LEG#16)

Legal ambiguity has also made it difficult to assign blame or responsibility when something goes wrong in online dispensing.

It is unclear who is responsible if a patient suffers harm from a medication purchased online. Without defined guidelines, accountability in cases of errors or misuse is ambiguous. (LEG#4)

Establishing a centralized regulatory framework is essential. It would provide clarity on accountability, ensure consistent enforcement of standards, and ultimately protect patients and pharmacists from potential legal and health risks. (LEG#17)

#### Panel of experts

To address these regulatory issues, many participants proposed the formation of a multidisciplinary panel to oversee policy development. This panel would ideally include pharmacists, legal experts, and IT specialists.

A group of multidisciplinary experts should be involved in regulating online pharmacies to ensure comprehensive oversight.” (MOH#9).

Such a collaborative approach was viewed as necessary for bridging gaps between technological, legal, and clinical considerations.

### Ethical considerations

Ethical concerns were central to participants’ responses, particularly in four areas: Prescription Forgery and Authenticity, Fair Access and Equity, Marketing and Advertising Practices, and Logistics and Distribution.

#### Prescription forgery and authenticity

Pharmacists raised serious safety concerns about the ease with which prescriptions could be forged in a digital setting. Participants called for more robust verification systems.

Online platforms often lack the mechanisms required to verify prescription authenticity, jeopardizing patient safety. (PH#5)

With advancements in AI and computing tools, forgery and document fabrication have become much easier. Ensuring the authenticity of prescriptions is a major challenge. (PH#11)

These insights underscore the urgent need for technological safeguards that can match or exceed those in brick-and-mortar pharmacies.

#### Fair access and equity

Participants noted that certain populations, such as the elderly, psychiatric patients, or minors, might be disadvantaged by online systems if equity is not proactively addressed.

Vulnerable groups, including minors, patients with psychiatric conditions, and elderly individuals with Alzheimer’s, require special support in online pharmacy programs as they may lack full decision-making capacity. (LEG#13)

Online pharmacies must adhere to principles of autonomy, non-maleficence, beneficence, and justice. (MOH#4)

Vulnerable populations may face challenges accessing these services, necessitating measures to promote equitable access. (MOH#15)

These statements indicate a consensus that digital equity should be foundational in online pharmacy design and regulation.

#### Marketing and advertising practices

Some participants expressed concern about aggressive or misleading marketing of medications online, which could violate ethical norms.

Illegal advertising can mislead consumers, highlighting the ethical dimensions of marketing practices in this field. (MOH#7)

We do not permit advertising for pharmaceutical products. (LEG#4)

The lack of clear boundaries around marketing contributes to public mistrust and misinformation risks.

#### Logistics and distribution systems

Participants proposed that logistics should be tightly integrated with licensed pharmacies to ensure safe and traceable distribution.

The preferred logistics model should belong to and be managed by the pharmacy itself to avoid reliance on public transportation services. (MOH#6)

If a pharmacy outsources logistics, it should partner with a licensed pharmaceutical distribution company with defined legal structures and technical oversight. (PH#11)

Instances of distribution through informal networks have sometimes resulted in medication being distributed outside the official system. (MOH#9)

These views reflect the need for professional and legally compliant logistics infrastructure in online medication delivery.

### The role of the pharmacist

Participants identified the pharmacist’s role as a cornerstone of safe and effective medication distribution, even in online contexts. Three key sub-themes were emphasized: Provision of Pharmaceutical Advice, Prescription Verification, and Medication Quality and Safety Assurance.

#### Provision of pharmaceutical advice

Pharmacists expressed concern that the digital environment may erode their traditional consultative role, reducing opportunities for patient interaction and education.

In online pharmacies, the face-to-face interaction between the pharmacist and the patient may be eliminated, which could lead to a loss of health-oriented recommendations and pharmaceutical information exchanges. (PH#12)

In an online setting, the pharmacist’s role as a healthcare provider might be reduced to merely a controller or supervisor. (MOH#9)

Online pharmacies often bypass essential guidance that we provide in face-to-face interactions, which can negatively impact patient education. (PH#2)

These comments reflect a shared belief that technological solutions must include channels for pharmacist-patient communication, such as chat functions or telepharmacy consultations.

#### Prescription verification

Participants stressed that safeguarding prescription accuracy is essential, even or especially in online settings. Pharmacists must remain central to this verification process.

Online platforms need a robust system for verifying prescriptions to ensure patients receive the correct medication for their conditions. (LEG#13)

Even in an online setting, pharmacists must maintain their consultative role. Patients require guidance on medication use, and pharmacists are essential for ensuring safety. (PH#2)

These statements highlight that human oversight remains crucial in validating prescriptions, regardless of digital advancements.

#### Medication quality and safety assurance

Pharmacists and online pharmacy managers highlighted the importance of controlling medication quality and safety during the sales and delivery process.

The quality of medications sold online must be as rigorously controlled as in physical pharmacies. Patients need to trust the products they purchase. (PH#1)

When a pharmacy sells a medication online and is responsible for delivering it to the patient’s home, additional safety measures must be taken to ensure the medication’s safety during transit. (PH#5)

This underscores the dual responsibility of ensuring product integrity, both in storage and delivery.

### Patient behavior and safety

Participants identified Patient Awareness and Education, Privacy and Confidentiality, and Consumer Confidence as key determinants of successful adoption of online pharmacy services.

#### Patient awareness and education

A lack of patient knowledge about how to safely navigate online pharmacies was seen as a significant barrier to trust and uptake.

Patients need greater awareness of the pros and cons of buying medications online. This could enhance trust and reduce misconceptions about online pharmacies. (PH#10)

Educational initiatives are necessary to inform patients about potential risks and promote safer practices. (MOH#9)

The emphasis here is on public education as both a protective measure and a trust-building strategy.

#### Privacy and confidentiality

Digital privacy was raised repeatedly as a core patient concern. Without guarantees of confidentiality, participants believed users would remain hesitant.

Without adequate security, patients may hesitate to use online pharmacy services. (PH#10)

Confidentiality and secure management of medications are critical. Patients must feel confident that their health information is safe, even during the delivery process. (MOH#9)

In medical law, autonomy or privacy is a core principle. It must be clear who has access to patient information. Is it only the individual preparing the prescription, or are there others? (LEG#4)

These perspectives illustrate that legal and technical infrastructure must jointly support strong privacy protection.

#### Consumer confidence

Trust was consistently described as a foundational element in patient willingness to use online services. The availability of branded options and transparent systems was seen as essential.

Establishing consistent quality standards and a reliable prescription verification system can significantly enhance trust in online pharmacies. (PH#11)

Patients trust in-person interactions more, especially for sensitive health issues. Online pharmacies must strive to build a similar level of trust. (PH#2)

Medications come from different brands—Iranian, Indian, or American. Each has its strengths and weaknesses, and prices vary. Online medication sales should allow patients to select their preferred brand via text or the platform. (LEG#13)

Together, these comments suggest that fostering autonomy and offering choice are integral to building consumer confidence in digital pharmaceutical services.

### Technology and infrastructure

This theme includes four sub-themes: Effective online platforms, Data security and Privacy Protection, and Internet Access and Digital Inequality. Participants emphasized the importance of efficient e-commerce platforms, robust data security, and equitable internet access.

#### Effective online platforms

Participants highlighted the role of advanced technology in creating efficient online pharmacy platforms to improve safety and sales efficiency. However, the lack of integrated systems was identified as a significant challenge.

Efficient and intuitive platforms are essential for bridging the gap between traditional services and online pharmacies, ensuring a seamless customer experience. (PH#12)

A secure and high-performance platform is needed for online pharmacies. Advanced technologies, especially AI, can be highly effective in designing and managing online pharmacies, including geolocation-based prescription fulfillment algorithms. (MOH#7)

Their insights point toward the need for investment in custom-built digital systems, not merely off-the-shelf solutions.

#### Data security and privacy protection

The importance of cybersecurity and patient data integrity was highlighted by all professional groups, particularly legal experts.

Online pharmacies must be accessible and user-friendly, with customer support services to guide patients through the process and answer any questions. (LEG#8)

Without a well-integrated electronic health system, ensuring safe and efficient sales is challenging. (PH#12)

Data security was a priority concern. Participants highlighted the risks of data breaches and emphasized the need for strong cybersecurity protocols to protect patients’ personal and medical information.

Cybersecurity is a primary concern. Without robust protections, online platforms are highly vulnerable to hackers, leading to severe privacy violations. (LEG#4)

The absence of strong data protection protocols not only jeopardizes patient privacy but also undermines trust in online healthcare services. (LEG#17)

These statements underscore the necessity of incorporating high-grade cybersecurity features and end-to-end encryption in any national platform.

#### Internet access and digital inequality

Several participants voiced concern that limited digital infrastructure, especially in rural areas, could exacerbate health disparities.

Digital inequality limits access to online pharmacies and affects the availability of essential medications for many patients. (MOH#6)

Internet issues, such as frequent outages or slow speeds in rural areas, compound the challenges of implementing online pharmacies. (PH#5)

The uneven distribution of medications favors urban centers over rural areas. While online pharmacies could enhance access, poor internet infrastructure in rural areas remains a barrier. (PH#3)

These remarks reveal the importance of equitable technology deployment as part of the broader healthcare reform effort.

### Economic implications

Economic implications were a major concern among participants, particularly regarding Pricing Structures, Drug Supply Chains, and Insurance Integration. These factors were seen as critical for ensuring both the sustainability of online pharmacies and the fairness of access for patients and traditional providers.

#### Pricing structures

Many participants noted that unregulated price competition from dominant online platforms poses a serious threat to traditional pharmacies. The affordability advantages for consumers may come at the cost of long-term market stability

Traditional pharmacies struggle to remain profitable when competing with larger platforms offering lower prices. (PH#3)

Economic disparities between pharmacies are anticipated in online medication distribution, as those with stronger digital and logistical capabilities will dominate the market. (MOH#9)

Pricing structures in online pharmacies should be fair to prevent harm to low-income patients. (PH#10)

These insights suggest that while digital platforms may introduce cost-saving opportunities, pricing oversight is needed to preserve market balance and consumer protection.

#### Drug supply chains

Online medication sales could significantly alter traditional pharmaceutical supply chains, affecting logistics, inventory management, and the reliability of medication distribution. Participants highlighted several potential disruptions.

The transition to online sales is transforming traditional supply chain processes. Disruptions in logistics and inventory management have raised concerns about the reliability of medication distribution. (PH#5)

Market monopolies by large companies could exacerbate challenges in ensuring medication authenticity and safety. (PH#11)

However, participants also recognized key advantages, such as improved access to medications, information about rare medication, optimized inventory management, and reduced patient travel.

The potential of online pharmacies to enhance access and reduce costs for patients is a recognized advantage. (PH#3)

The main benefits of online medication delivery platforms include ease of access, information about rare medications, optimized inventory management, and reduced urban travel. (MOH#9)

These perspectives reflect the dual-edged nature of digital transformation in the supply chain: while it can offer major improvements in efficiency and access, it also requires careful design to avoid unintended harms.

#### Insurance integration

Integration of online sales with insurance systems was seen as a key step toward equitable access and financial sustainability. Participants pointed out that most online pharmacies currently operate outside insurance coverage, creating affordability challenges.

Insurance should cover online prescriptions to make them affordable, just like traditional pharmacies. (PH#1)

High treatment costs are a major concern for patients seeking to reduce expenses. Connecting online pharmacy platforms with insurance systems would provide comprehensive, real-time information and enhance convenience. (MOH#7)

Integration with insurance systems allows patients to access safe and fairly priced medications without the hassle of reimbursement or searching for pharmacies that accept specific insurance plans. (MOH#7)

These responses indicate strong support for policy reforms that bridge the gap between traditional and digital care financing, ensuring that online platforms become a supplement to, rather than a burden on, existing health systems.

Across all six themes, participants consistently emphasized the need for integrated, ethically sound, and technologically robust policies to guide the expansion of online pharmaceutical services in Iran. Their insights revealed both the potential benefits, such as improved access, cost-efficiency, and supply chain optimization, and the risks, including legal ambiguity, ethical dilemmas, and inequities in digital access. The quotes reflect strong stakeholder consensus on the urgent need for policy clarity, equity considerations, and infrastructure investment to support safe and effective implementation.

## Discussion

The findings from this study highlight several critical factors influencing the online sale of prescription medications in Iran, aligning with and expanding upon existing global literature on the opportunities and challenges of e-pharmacy systems. The discussion is organized around the six main themes identified: legal and regulatory challenges, ethical considerations, the role of the pharmacist, patient behavior and safety, technology and infrastructure, and economic implications.

The absence of clear legal frameworks was a central theme among participants, with concerns about regulating online pharmacies, preventing counterfeit medications, and ensuring accountability. This finding is consistent with international studies, where the absence of national e-pharmacy regulations has hampered safe digital medicine distribution [19]. Moreover, previous research on e-health services has noted the difficulties in adapting existing healthcare regulations to digital environments, stressing the need for new frameworks that address online medication distribution specifics [20]. In Iran, the unique regulatory and cultural landscape further complicates the adoption of online pharmacy systems, suggesting a need for legal reforms tailored to local needs.

Participants’ emphasis on patient data security, prescription forgery, and the protection of vulnerable groups aligns with global concerns [21, 22]. Recent reviews have warned that ethical lapses in digital health can erode public trust [23]. Protecting vulnerable groups, such as the elderly or those with psychiatric conditions, also requires additional measures to prevent exploitation and ensure fair access. This echoes findings of Tiffin et al. [24], who noted the risks to vulnerable populations in digital healthcare services without strong ethical oversight.

The shift in the pharmacist’s role from direct interaction to digital mediation presents a significant challenge. Recent international case studies from countries with advanced e-pharmacy systems have shown that virtual pharmacist consultations improve patient outcomes when integrated into online platforms [25, 26]. These findings validate the participants’ suggestion that pharmacist-patient communication tools—such as live chat, video consultations, or AI-based triage—are essential for maintaining therapeutic relationships and medication adherence.

Trust in online pharmacies remains a pivotal issue. Research indicates that patient trust in e-pharmacies often hinges on visible data protection measures and accessible support services [27]. As online pharmacies introduce new dynamics in patient-provider relationships, studies emphasize that ensuring transparency, respecting patient privacy, and delivering reliable services are essential for patient adoption [28]. Additionally, public education on the safe use of online pharmacies could help address existing knowledge gaps, as evidence shows that consumer awareness programs can significantly enhance public perceptions and promote safer usage practices [29]. These findings reinforce this study’s recommendation to invest in targeted public education campaigns that clearly communicate the risks and benefits of e-pharmacy services.

Participants expressed that a robust technological infrastructure is vital for supporting e-pharmacy services. This finding is corroborated by studies indicating that efficient, interconnected platforms are crucial to managing inventory, verifying prescriptions, and preventing data breaches [30]. Moreover, studies have shown that AI-powered prescription verification tools and blockchain-enabled supply chains can improve security and efficiency [31]. The literature underlines that incorporating AI, secure electronic health records, and cybersecurity protocols is more likely to succeed in protecting patient data and enhancing operational efficiency [32]. However, the current absence of a unified national electronic health database in Iran reflects broader infrastructural deficiencies and remains a significant issue.

The economic disruption posed by online pharmacies, particularly their impact on traditional brick-and-mortar pharmacies, was a major concern among participants. They proposed that integrating online medication sales with insurance reimbursement mechanisms and implementing fair pricing policies could help mitigate competitive imbalances. Supporting this view, evidence indicates that extending insurance coverage to online purchases can level the playing field and significantly reduce out-of-pocket costs for patients [33]. Such measures would facilitate the coexistence of traditional and online pharmacies, while also enhancing accessibility and affordability of medications, ultimately benefiting the broader healthcare system.

### Future research

Future research should examine these findings quantitatively and incorporate patient perspectives to provide a more comprehensive understanding of the success factors driving online pharmacy adoption in Iran. In practical terms, developing secure digital platforms, ensuring equitable access, and integrating with insurance systems are crucial for improving service delivery and affordability.

### Limitations of the study

The online pharmaceutical industry in Iran is relatively new and rapidly evolving, with a legal and regulatory landscape subject to frequent changes. As a result, some insights shared by participants in this study may quickly become outdated or less relevant as new policies emerge. Moreover, consumer perspectives were not included, which limits the understanding of patient trust, preferences, and behavior critical factors for the adoption of online pharmacy services.

Although a purposive sampling strategy was employed to include participants with relevant expertise, some degree of convenience sampling may have influenced participant recruitment, particularly due to accessibility and existing professional networks. This may have limited the inclusion of less accessible or dissenting perspectives. Future research should aim to incorporate broader recruitment strategies, patient views, and quantitative methodologies to validate and expand on these findings.

## Conclusion

This study highlights the complexities of implementing online prescription medication sales in Iran, underscoring the need for clear legal and regulatory frameworks. Establishing a panel of experts, including pharmacists, IT specialists, and legal professionals, could ensure the development of effective regulations. Additionally, designing a nationwide, user-friendly online medication sales platform with integration capabilities for health management systems, electronic prescriptions, and insurance platforms is crucial. The platform must prioritize robust technical infrastructure, data security, and patient privacy protection while addressing preventive measures against malpractice. By meeting these requirements, online prescription sales can be effectively implemented, ensuring safety, equity, and transparency.

## Supplementary Information

Below is the link to the electronic supplementary material.

Supplementary Material 1

Supplementary Material 2

## Data Availability

The datasets used and/or analysed during the current study are available from the corresponding author on reasonable request.

## Abbreviations

AI: Artificial Intelligence
COREQ: Consolidated Criteria for Reporting Qualitative Research
eHealth: Electronic Health
HIE: Health Information Exchange
IT: Information Technology
MAXQDA: MAX Qualitative Data Analysis (software)
OTC: Over-the-Counter
TUMS: Tehran University of Medical Sciences
PH: Pharmacist
LEG: Legal Expert
MOH: Policymaker
