# Supplementary material for: Factors influencing the online sale of prescription medication in Iran: insights from a qualitative content analysis
Source: BMC Health Serv Res. 2026 Aug 18;26:1604. doi: 10.1186/s12913-026-15338-9 (PMC13761082; doi:10.1186/s12913-026-15338-9)
Supplement: Supplementary file 2 [file 12913_2026_15338_MOESM1_ESM.docx]

**Interview Guide**

**Description**: "English version of the semi-structured interview guide used to explore factors influencing the online sale of prescription medications in Iran."

**Introduction:**

Thank you for participating in this study. The purpose of this interview is to explore the "**Factors Influencing the Online Sale of Prescription Medications in Iran**." Your insights will contribute to understanding the challenges and opportunities associated with the creation and development of online pharmacies in Iran.

- This is a semi-structured interview. I will ask general questions, but please feel free to elaborate on your answers and share your thoughts openly.
- Your interview is recorded and your responses will remain confidential and will only be used for the purposes of study.
- Your participation is voluntary. You may decline to answer any question or withdraw from the interview at any time. Your responses will be anonymized to protect your identity.

**Background Question**

- Please introduce yourself and explain your professional background (e.g., pharmacist, legal expert, policymaker), particularly your involvement in the field of online sales of prescription medications.

**Main Questions**

1. Could you share your experience with the process of distributing prescription medications through online pharmacies?
2. From your perspective, what are the most significant:
   - **Dvantages?**
   - **Disadvantages** (challenges or concerns)?
   - **A Threats** related to the online sale of prescription medications in the country?
3. How effective are the current processes and systems used by online pharmacies to ensure:
   - **Patient safety?**
   - **Accuracy in dispensing medications**?
4. What solutions would you suggest to address the challenges in the online medication distribution process?
5. How can public awareness and trust in online pharmacies be improved?"
6. In your opinion, what role can technology and interdisciplinary collaboration play in Improving the overall online pharmacy system?
7. What technological or infrastructural changes do you think are necessary for the safe and effective sale of prescription medications online?

**Additional Recommendations:**
Do you have any other suggestions or recommendations to improve this research or the online sale of prescription Medications?

**Conclusion of the Interview**

Is there anything else you would like to discuss regarding the online sale of prescription medications in Iran that we haven’t covered in this interview?

Thank you for your time and valuable insights. Your participation is crucial to this research and will help shape the future of online pharmacies in Iran.
